# Percolative Composites with Carbon Nanohorns: Low-Frequency and Ultra-High Frequency Response

**DOI:** 10.3390/ma12111848

**Published:** 2019-06-06

**Authors:** Olga V. Sedelnikova, Kseniya I. Baskakova, Artem V. Gusel’nikov, Pavel E. Plyusnin, Lyubov G. Bulusheva, Alexander V. Okotrub

**Affiliations:** 1 Nikolaev Institute of Inorganic Chemistry SB RAS, 3 Acad. Lavrentiev Ave., 630090 Novosibirsk, Russia; baskakova@niic.nsc.ru (K.I.B.); artemg@ngs.ru (A.V.G.); plus@niic.nsc.ru (P.E.P.); bul@niic.nsc.ru (L.G.B.); spectrum@niic.nsc.ru (A.V.O.); 2 Laboratory for Terahertz Research, Tomsk State University, 36 Lenin Ave., 634050 Tomsk, Russia

**Keywords:** carbon nanohorns, DC conductivity, AC conductivity, permittivity, electromagnetic shielding

## Abstract

We systematically studied the electromagnetic properties of carbon nanohorns (CNHs) and polystyrene composites filled with CNHs in static regime, low frequency and microwave regions. CNHs were synthesized using the direct current arc-discharge method using solid graphite rods and graphite rods filled by melamine mixed with graphite powder. Transmission electron microscopy and thermo-gravimetric analysis showed that CNH agglomerates are the main product, while the addition of melamine promotes the formation of graphite balls. Graphitic contamination causes the internal leakage of inter-agglomerate capacity, lowering the permittivity and enhancing the conductivity of composites. The permittivity of CNH/polystyrene composites increases with the filler fraction, and near the dielectric threshold electromagnetic characteristics of the composites exhibit critical behaviour. Our results suggest that CNHs with relatively high values of permittivity and contact resistance could be used as high-*k* materials.

## 1. Introduction

Today, polymer composites with conductive particles are an actively explored field. In particular, the near-percolated composites with carbon nanostructures are promising for fabrication of high-*k* materials [1,2]. The combination of conductive nanoparticles and very thin polymer layers in between provides an anomalously high dielectric constant for the material [1,3]. In the vicinity of the percolation threshold and below it, the dielectric constant of composites containing 10 wt % of randomly dispersed carbon nanotubes showed a dielectric constant of approximately 50, while the loss tangent was approximately 0.4 [4]. Similar properties were observed for the composite containing about 4 wt % of graphene oxide [5]. Comparison of the dielectric properties of thin polymer films with different types of carbon nanostructures (carbon nanotubes and nanofibers, graphene nanoplatelets) showed the higher permittivity and conductivity when using wire-shaped structures [6]. The spherical inclusions, such as carbon black [7] and onion-like carbon [8], showed the less impressive responses due to lower conductivity and less effective composite filling.

Carbon nanohorns (CNHs) are nanotube-like structures with conical tips and a typical diameter of about several nm [9,10]. Forming as roughly spherical aggregates of short conductive graphitic tubular particles [11,12,13,14], CNHs occupy an intermediate position between wire-shaped and spherical carbon nanostructures. Therefore, they could be attractive for electromagnetic applications. According to the data reported, the conductivity of CNHs could vary from 10^−3^ S/m [15] to several hundred S/m [16,17], which is probably caused by the different densities of the materials. Addition of CNHs to dielectric or conductive polymer increases the conductivity slightly [18,19,20,21,22,23]. The permittivity and microwave shielding effectiveness (SE) of the materials with CNHs have barely been investigated. We have found just a few works devoted to three-component polymer composites, where the second filler was presented by graphene [18,19], ferromagnetic particles [20] or their hybrids [21,22] in addition to the CNHs.

In this report, we systematically investigate the electromagnetic properties of CNHs synthesized using the arc discharge method. We found that the addition of melamine into the electric arc affects the density and composition of the synthesis product. For polystyrene (PS) composites with high CNH loading the dielectric and conductive percolation-like critical behaviors were observed.

## 2. Materials and Methods

### 2.1. Materials

The setup for arc discharge synthesis is described in detail elsewhere [24]. The principal scheme is shown in Figure S1 (Supplementary Materials). Briefly, an upper movable cathode was made from a water-cooled graphite rod of 60 mm diameter. A graphite anode was used which had a cross-section of 14 × 14 mm and a length of 200 mm. The anode was placed vertically on a water-cooled holder in bottom of the chamber opposite the cathode. All syntheses were carried out at a direct current of 500 A, a voltage of 50 V and a helium pressure of 5 × 10^4^ Pa.

CNH samples were collected from a metallic screen placed between the evaporated electrode and water-cooled walls of the chamber. The initial weight of the anode was *ca*. 70 g and the mass of the soot deposited on the screen was *ca*. 10 g. Evaporation of the pure graphite rod produced the sample denoted as S0. Previously we found that the addition of foreign components in the electric arc strongly modifies the morphology and electromagnetic properties of product [25,26,27]. In this work, we altered the arc discharge process by the addition of melamine. In particular, a cylindrical cavity in the central part of the rod was filled with melamine mixed with graphite powder. The concentration of melamine was about 2 and 4 wt % of the weight of the evaporated rod. The samples obtained after the syntheses are denoted as S2 and S4, respectively. The duration of the syntheses of samples S0 and S2 was 30 min. When 4 wt % of melamine was added, the anode was consumed after 10 min.

### 2.2. Preparation of CNH/PS Composites

The procedure of preparation of PS composites with carbon nanomaterials is described in our previous works [8,28,29,30,31]. PS and CNHs were mixed in 20 mL of chloroform under a sonication treatment (30 W) for 30 min. In general, the fast drying of such suspensions produces highly porous polymer samples. To avoid air bubbles in film, the CNH/PS suspensions were dried in a fume hood at room temperature for 1 day. The content of CNHs was varied from 1 to 32 wt %, from 1 to 24 wt % and from 1 to 30 wt% for S0/PS, S2/PS, and S4/PS composites, respectively. The further increase in the filler loading gave fragile samples. Note, that the CNH manufacturing threshold was between the values for composites with carbon nanotubes and carbon nanofibers (~2 wt %) [6] and carbon onions (~35–40 wt %) [8,28]. As a result, we obtained thin plates with a thickness of 80–150 μm for static and low-frequency measurements and 280–550 μm for microwave measurements. All composites obtained had a visually uniform color from dark grey to black depending on the filler content.

### 2.3. Methods

The structure of carbon nanomaterials was studied using transmission electron microscopy (TEM) on a JEOL 2010 microscope (JEOL Ltd., Tokyo, Japan). The images were obtained at 200 kV accelerating voltage. Raman spectra were collected on a Spex 1877 triple spectrometer (Spectroscopy & Imaging GmbH, Warstein, Germany) using the 488 nm line from an argon laser. Thermo-gravimetric analysis (TGA) in Ar/O_2_ (80:20 wt%) atmosphere was done on a STA 449 F1 Jupiter thermal analyser (Netzsch, Selb/Bayern, Germany) in Al_2_O_3_ crucibles (900 °C max, 10 K/min).

The direct current (DC) conductivity *σ*_DC_ measurements of rectangular-shaped samples (1.2 × 1.2 cm) were carried out using a four-probe technique using an R3009 measuring bridge. Electrical contacts were made with silver glue.

The real and imaginary components of the impedance were measured using a two-contact method on a Bio-Logic SP-300 potentiostat (Bio-Logic Science Instruments SAS, Seyssinet-Pariset, France) in a frequency range of 1 kHz–7 MHz. For measurements, composite samples with a size of ca. 1.2 × 1.2 cm were used. The frequency dependences of the alternating current (AC) dielectric constant *ε*_AC_ and conductivity *σ*_AC_ were recovered from the impedance using the method described in [8].

Microwave measurements of composite materials were carried out using a Mikran scalar network analyzer P2M-04A (Mikran, Tomsk, Russia) in the Airline coaxial line 12.93 mm in the range of 1–4 GHz using the standing wave ratio sensor (SWR) DK1-04-11P-11R (Mikran, Tomsk, Russia). Composite samples for the measurements were ring-shaped with an inner diameter of *ca*. 3 mm and an outer diameter of *ca*. 7 mm. In the result, the powers of the wave reflected from the sample surface (*S*_11_) and the wave passing through the sample (*S*_21_) were obtained. Transmittance (*T*), reflectance (*R*), and absorbance (*A*) were determined from *S*_21_ and *S*_11_ values for 1 cm thickness as: *T* = (*S*_21_)^2^, *R* = (*S*_11_)^2^, and *A* = 1 − *T* − *R*.

## 3. Results and Discussion

### 3.1. Structural Characterisation

**Figure 1:**
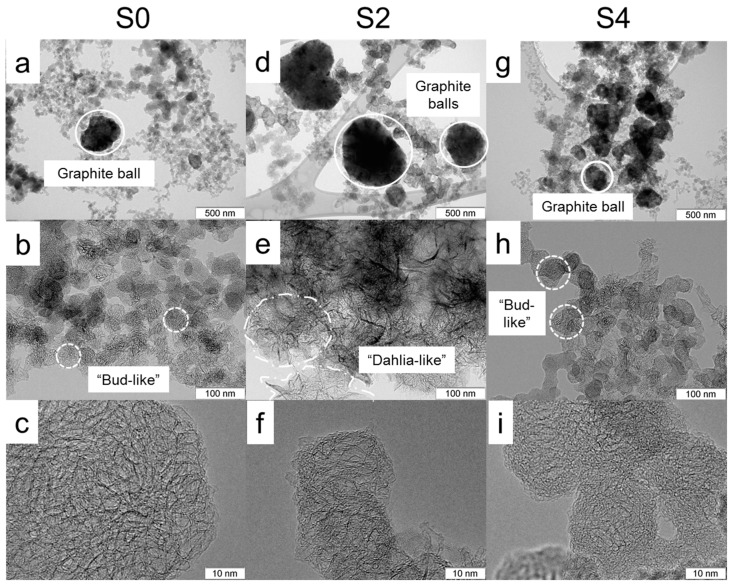
Typical transmission electron microscopy (TEM) images of S0 (**a**–**c**), S2 (**d**–**f**), and S4 (**g**–**i**) samples.

Figure 1 shows typical TEM images of CNH samples. Dark, big-size quasi-spherical particles are composed of aggregated graphitic layers, which are usually called graphitic balls (GBs). According to TEM study, the portion of GBs was larger for the S2 and S4 samples than for the S0 sample. It has been proposed that formation of GBs is determined by temperature gradient during the arc burning [12]. It is likely that the melamine additive in the evaporated rod influences this parameter, promoting the formation of GBs. Arc-discharge synthesis involves many chemical and physical acts and there is no guarantee that the variation of any synthesis parameter provides a linear effect on the structure of the product. This is what we observed in our case. An increase in the melamine content in the evaporated graphite rod affects the density of the CNHs powder nonlinearly. The average CNHs density measured with a pycnometer was 0.26, 0.13, and 0.64 g/cm^3^ for S0, S2, and S4 samples, respectively. The lower density of S2 CNHs correlates with lower mixing weight threshold for S2/PS (24 wt %), implying the higher surface area and the harder viscosity issues in nano-reinforced composite preparation. The smaller, soot-like particles are agglomerates of CNHs, consisting of one- or few-walls. The S0 sample mainly contains the particles from tightly packed “bud-like” CNHs. Addition of melamine results in the formation of less dense CNH agglomerates, which are usually called “dahlia-like” agglomerates. Due to a complex shape of the boundaries of these agglomerates, we can only estimate their size using the TEM images of several distinguishable individual particles. The average size of “dahlia-like” agglomerates was about 80 nm in the S2 sample and about 60 nm in the S4 sample. The mean size of “bud-like” agglomerates decreased from 45 nm in the S0 sample to 27 nm in the S4 sample (Figure S2, Supplementary Materials). Moreover, single- and few-layered graphene can be detected in the S2 and S4 samples, respectively (Figure S3, Supplementary Materials). According to our previous work, nitrogen present in the arc discharge can promote formation of graphene layers [27].

**Figure 2:**
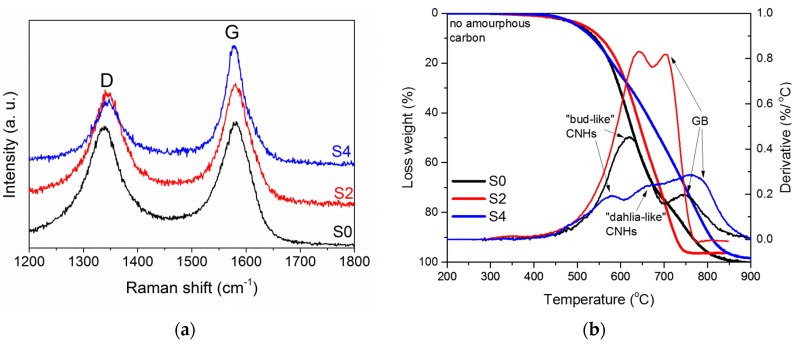
Raman spectra (**a**) and TG and DTG curves (**b**) for S0, S2, and S4 samples.

Raman spectra of CNH samples exhibited two peaks at 1340 cm^−1^ (D band) and 1580 cm^−1^ (G band) (Figure 2a), which are typical for *sp*^2^-hybridized carbon nanomaterials [32]. Addition of melamine in the evaporated graphitic rod results in a decrease of the intensity of the D band, indicating a more perfect structure of particles in the S2 and S4 samples in comparison to those in the S0 sample (*I*_D_/*I*_G_ = 0.98 for S0 and 0.78 for S2 and S4). We suggest that the decrease in the *I*_D_/*I*_G_ ratio is mainly due to a large fraction of GBs in the S2 and S4 samples, as TEM images showed (Figure 1).

The TGA of the samples detected no weight loss up to 500 °C (Figure 2b). This is evidence of the absence of amorphous carbon in our samples. The differential thermogravimetric (DTG) curve of the S0 sample had two peaks at ca. 620 and 750 °C, which correspond to the burning of single-walled “bud-like” CNHs [12,33,34] and graphitic particles [12,33]. The S2 sample also burned in two stages. Temperatures of 645 °C and 705 °C could be attributed to “dahlia-like” CNHs and GBs/graphenes, respectively. The DTG curve of the S4 sample had three peaks at 580 °C, 657 °C and 770 °C, which could be ascribed to the burning of “bud-like” CNHs, “dahlia-like” CNHs, and graphitic by-products. The TGA data show that CNHs are the primary phase in all samples and addition of melamine in the reaction decreased their content in the synthesis product. This result agrees with a very low density of the S0 and S2 samples. A larger density of the S4 sample is due to the notable content of graphitic particles, as TGA determined.

### 3.2. Electromagnetic Properties of CNHs

**Figure 3:**
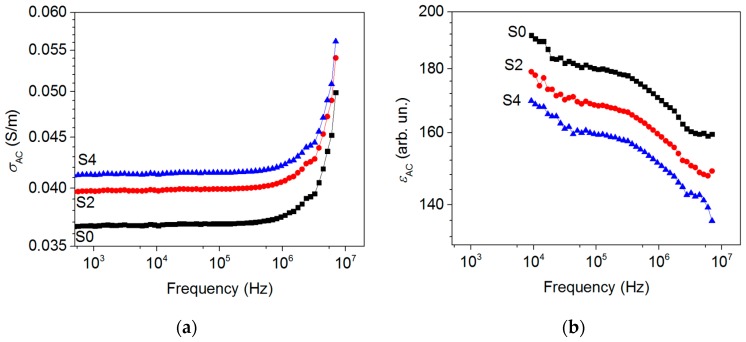
*σ*_AC_ (**a**) and *ε*_AC_ (**b**) of S0, S2, and S4 samples.

Figure 3 shows the log-log frequency dependence of the conductivity *σ*_AC_ and the permittivity *ε*_AC_ measured at room temperature for the CNH powders. Above 1 MHz, the conductivity increases following a power law behaviour known as “universal dynamics response” [35]. As a result, *σ*_AC_(*ω*) can be expressed as

*σ_AC_*(*ω*) = *σ*_DC_ + *A ω^s^*, (1)

where *σ*_DC_ corresponds to the direct current conductivity, which can be estimated from the low-frequency plateau in the *σ_AC_*(*ω*)-*ω* plot, *s* = 1. The S0 sample had a DC conductivity of 0.036 S/m, whilst the samples obtained with the addition of melamine had slightly higher *σ*_DC_ values (0.040 and 0.041 S/m for S2 and S4 samples, respectively). The AC permittivity drops with frequency. At 1 MHz, the permittivity of the S0 sample is approximately 180. This characteristic decreases to 170 for the S2 sample and 160 for the S4 sample. The relatively high value of the CNH permittivity agrees with extremely low densities of CNHs, implying that our samples can considered weakly bounded porous agglomerates. As a result, the permittivity component has a primary role due to its interparticle capacity contribution, while conduction through the sample is restricted by air gaps.

### 3.3. DC Conductivity of CNH/PS Composites

**Figure 4:**
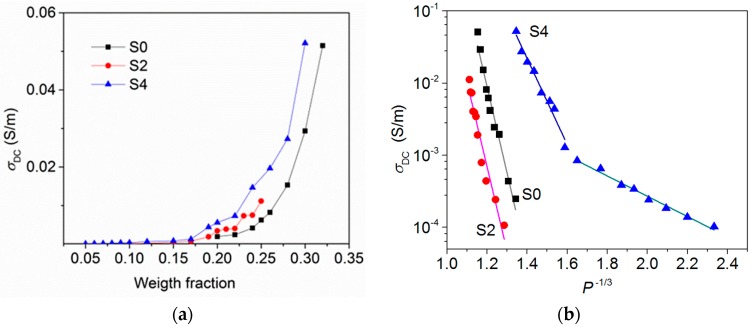
*σ*_DC_ as the function of filler weight fraction (**a**) and log *σ*_DC_ as the function of filler volume fraction (**b**) measured for polystyrene (PS) composites with S0, S2, and S4 powders.

The DC conductivity of the PS composites with the S0, S2, and S4 powders is shown in Figure 4 as a function of the weight fraction of the filler. *σ*_DC_ is negligible for filler content below 20 wt %. With a further increase in the filler loading, the conductivity of the composite increases. Above the percolation threshold DC conductivity should have a plateau corresponding to the electrical response of a percolating network. Such behavior was observed for the composites with a low contents of anisotropic fillers (graphene [36], carbon nanotubes [37,38], and carbon nanofibers [39]) and a rather high concentration of onion-like carbon (about 7–10 wt %) [40]. Our data show that even for the composites with maximum possible loading (manufacturing threshold), the DC conductivity does not reach the constant value. It indicates a high interfacial resistivity, which suppresses tunneling between adjacent CNHs.

The DC conductivity decreases as *P*^−1/3^ (Figure 4b), where *P* is the volume fraction of filler in the composite calculated from the weight fraction using the CNHs density. This means that the nature of contacts between CNH agglomerates embedded in the PS matrix can be attributed to tunneling through potential barriers [41]. It was shown that the tunneling mechanism determines transport through composites with carbon black [42], carbon nanotubes [37], and onion-like carbon [43]. The linear regions cross each other when the content of S4 sample is about 17 wt %. This behaviour could be due to the presence of GBs, which are many in the S4 sample, and they also should contribute to the conduction path for the highly filled S4/PS composite. For the S0/PS sample with 30 wt % filler loading and S4/PS sample with 28 wt % filler loading, the DC conductivity is about 0.05 S/m, that is, larger than the *σ*_DC_ values for the S0 and S4 powders found from low-frequency *σ*_AC_ plateaus (Figure 3a). This could be due to a partial breaking of CNH agglomerates during composite fabrication. As the result, the interparticle distance in the composite decreases in comparison with the value in powder, simplifying the electron hopping between conductive inclusions.

### 3.4. AC Permittivity and Conductivity of CNH/PS Composites

**Figure 5:**
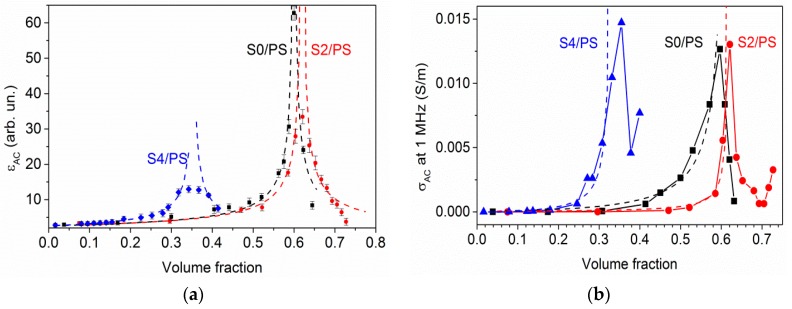
*ε*_AC_ (**a**) and *σ*_AC_ (**b**) of carbon nanohorn (CNH)/PS composites at 1 MHz. Dashed lines are the fitts of experimental data by Equations (2) and (3).

Figure 5 shows the AC permittivity *ε*_AC_ of composites containing 1–32 wt % of CNHs at 1 MHz. The *ε*_AC_(*ω*) values measured in the 1 kHz–7 MHz frequency range are collected in Figure S4 (Supplementary Materials). The permittivity of the free matrix is featureless across the frequency range shown and has a constant value of about 2.6, which is close to the expected value for dense bulk PS. This means that the method used for preparation of composite samples avoided the appearance of additional air bubbles, which increase the sample’s porosity. The conductivity of the free matrix (*σ*_PS_ ~ 10^−9^ S/m for 1 MHz) is negligible in comparison with the values for CNH powders.

At 1 MHz, the permittivity of S0/PS, S2/PS, and S4/PS composites increases with the filler loading, and at a critical value *P*_C_ (59, 62, and 33 vol%, respectively) it reaches the maximum values of 62, 34, and 13, respectively (*ε*_AC_ = 300, 58 and 55 at 1 KHz, see Figure S4). Note that the PS-based composites with quasi-spherical onion-like carbon showed the percolation threshold near 20 wt% [8,29] that is close to the value for CNH-based composites. However, the addition of CNHs, which is more conductive than onion-like carbon, enhanced the permittivity more. Further increase of the CNH content decreased the permittivity to approximately 8, 4 and 7 for the S0/PS, S2/PS, and S4/PS samples, respectively. A high permittivity and a large tunneling resistivity of CNH/PS composites suggest that CNHs are promising for use in the fabrication of high-*k* materials.

In general, the permittivity diverged as *P* is approached from either side:

*ε*_AC_ = *ε^PS^* |*P* − *P*_C_|^−*t*^, (2)

where *t* is the critical exponent. Fitting of the data measured by Equation (2) gives *t* = 0.61 for composites with S0 and S2 fillers and *t* = 0.35 for S4/PS samples (dashed lines in Figure 5a). These values, lower than the universal value for three-dimensional percolating systems (*s* = 0.8) [44], can mean that the percolation takes place in a network displaying more “dead arms” than classical random network. The reason for critical behaviour (2) for the permittivity is the existence of network of inter-agglomerate microcapacitors. The higher effective electric field that develops around the closely-packed agglomerates separated by the PS layer can be the cause of the critical increase in permittivity in the CNH/PS composites. When filler content is above the critical loading *P*_C_, hopping conduction through bulk samples occurrs. Internal leakage of the capacitors results in lower permittivity.

For low filler content, *σ*_AC_ of CNH/PS samples demonstrates PS-like dispersion (Figure S4). Starting from 10 wt % of the S0 sample and 12 wt % of the S2 and S4 samples, it follows a power law dependence similar to that of the CNH powders. Fitting of *σ*_AC_ with Equation (1) gives the *σ*_DC_ and *s* values listed in Table 1. One can see that general trends of the *s* parameter obtained and volume fraction *P* agree with each other.

Near the dielectric threshold, the composites also show AC conductivity critical behavior with the maximal conductivity of approximately 0.012 S/m for S0/PS and S2/PS samples and 0.015 S/m for the S4/PS sample (Figure 5b). Below this loading, the conductivity can be approximated within the generalized effective medium equations for binary systems [45]:

(1 − *P*) (*σ*_PS_^1/*u*^ − *σ*_AC_^1/*u*^)/(*σ*_PS_^1/*u*^ + *Aσ*_AC_^1/*u*^) + *P* (*σ*_CNH_^1/*u*^ − *σ*_AC_^1/*u*^)/(*σ*_CNH_^1/*u*^ + *Aσ*_AC_^1/*u*^) = 0, (3)

where *σ*_CNH_ is conductivity of CNHs at 1 MHz, *A* is a volume fraction-dependent coefficient, and *u* is the fitting parameter (*u* ≈ 1 for all samples).

A further increase in the CNHs content decreases the *σ*_AC_ value. We suggest that the amount of PS is not sufficient to bind the porous filler at CNH contents above 58, 62, and 32 vol% of S0, S2, and S4, respectively. As a result, the CNH agglomerates would not be kept tight enough to transfer electrons and, thus, the electrical conductivity of the composite would be decreased. The similar behavior of *σ*_AC_ of highly filled polymer composites is in agreement with the previous works [46,47].

### 3.5. Electromagnetic Shielding Effectiveness of CNH/PS Composites

**Figure 6:**
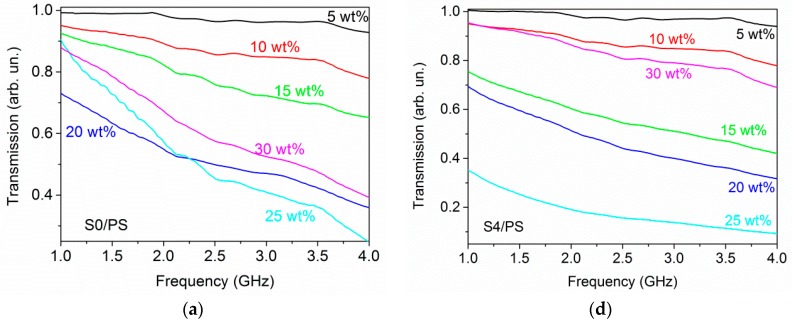

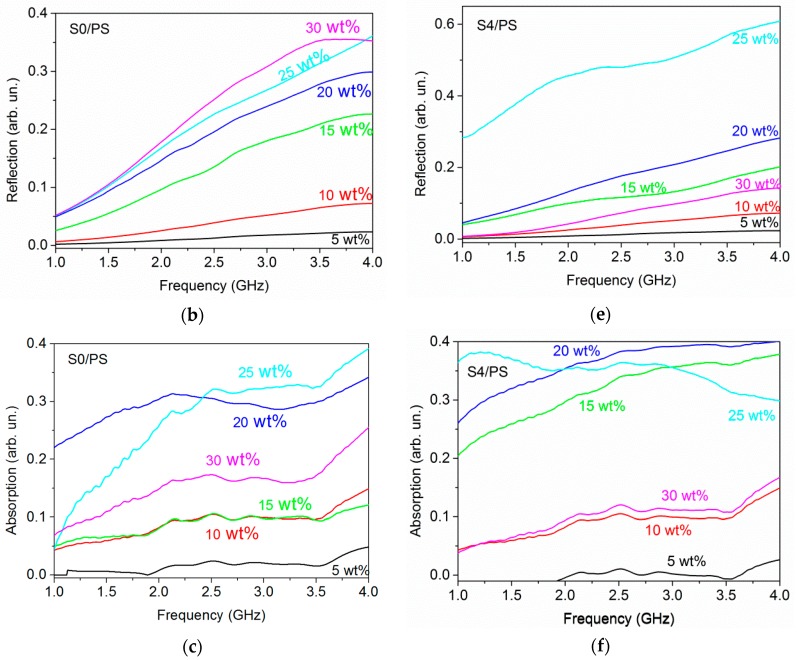
Transmission, reflection, absorption of S0/PS (**a**–**c**) and S4/PS (**d**–**f**) composites.

**Figure 7:**
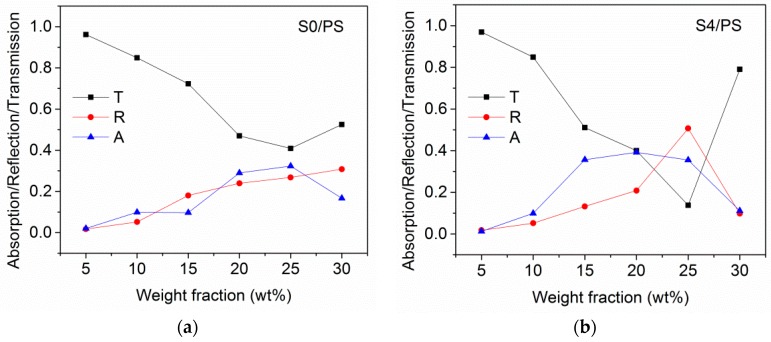
Transmission, absorption, and reflection of S0/PS (**a**) and S4/PS (**b**) composites at 3 GHz.

Microwave transmission *T*, reflection *R*, and absorption *A* of S0/PS and S4/PS composites are shown in Figure 6. The samples with 5 and 10 wt % of CNHs loading are almost opaque. Increase of the filler concentration reduces the transmittance significantly. For S0/PS samples containing 5–25 wt% of filler, the electromagnetic attenuation ability is due to equivalent contributions from reflection and absorption mechanisms (Figure 7a), while the reflection becomes dominant for the composite with 30 wt % of CNHs, which is above the critical loading fraction observed from AC measurements (~28 wt %, Supporting Information). A further increase in the content of S0 powder slightly decreases the transmission. This effect is more pronounced for S4/PS samples where the *T* value changes from 13% for 25 wt % loading to 79% for 30 wt % loading (Figure 7b). At that, the reflection displays a reverse trend at the same S4 content, indicating the dominant role of inter-agglomerate conduction paths in attenuation ability of CNH/PS composites.

## 4. Conclusions

This work aimed to expand the poorly studied field of CNHs. CNHs were synthesized by an arc-discharge of graphite rod without or with the addition of melamine. SEM and TGA data confirmed the formation of nanohorn agglomerates. At 1 MHz, the CNH powders showed relatively high permittivity (160–180) and rather low conductivity (*ca*. 0.04 S/m) indicating the primary role of micropacacity behavior of neighbor CNH agglomerates. The addition of melamine: (1) unbalances the electric arc, resulting in the tearing of graphitic fragments; and (2) affects the temperature gradient, preventing the formation of CNHs that gain up to 30 wt % of graphite particles in the product. Graphitic contaminations cause an internal leakage of inter-agglomerate capacity, lowering the permittivity and enhancing the conductivity of CNH/PS composites. The percolation-like critical behaviours were observed for low-frequency permittivity and conductivity, as well as for the microwave electromagnetic response of composites.

## Figures and Tables

**Table 1:** Parameters describing charge-transport properties of CNH/PS composites below the percolation threshold.

S0/PS				S2/PS				S4/PS			
*p*, wt %	*σ*_DC_, S/m	*s*	*P*, vol%	*p*, wt %	*σ*_DC_, S/m	*s*	*P*, vol%	*p*, wt %	*σ*_DC_, S/m	*s*	*P*, vol%
10	6∙× 10^−5^	0.11	31	10	2∙× 10^−5^	0.31	47	12	4 × 10^−5^	0.16	18
15	6 ×∙10^−4^	0.24	41	15	1 × 10^−4^	0.44	59	15	1 × 10^−4^	0.24	22
17	2∙× 10^−3^	0.35	45	16	5 ×∙10^−3^	0.56	60	19	7 × 10^−4^	0.25	28
20	3∙× 10^−3^	0.39	50	17	1 × 10^−2^	0.58	62	20	3∙× 10^−3^	0.32	29
25	8∙× 10^−3^	0.51	57					24	1 × 10^−2^	0.34	34
27	1 × 10^−2^	0.61	60								

## Supplementary Materials

The following are available online at https://www.mdpi.com/1996-1944/12/11/1848/s1, Figure S1: Principal scheme of arc-discharge setup; Figure S2: Histograms of the size distribution of CNHs; Figure S3: TEM images; Figure S4: AC permittivity and conductivity for composites containing in 1 kHz–7 MHz frequency range.
