# Supplementary material for: Percolative Composites with Carbon Nanohorns: Low-Frequency and Ultra-High Frequency Response
Source: Materials (Basel). 2019 Jun 6;12(11):1848. doi: 10.3390/ma12111848 (PMC6600986; doi:10.3390/ma12111848)
Supplement: Supplementary file 1 [file materials-12-01848-s001.pdf]

materials

*Type of the Paper (Article, Review, Communication, etc.)*

# Supporting Information for

# Percolative Composites with Carbon Nanohorns: Low-Frequency and Ultra-High Frequency Response

**Olga V. Sedelnikova [1,2,*], Kseniya I. Baskakova [1], Artem V. Gusel'nikov [1], Pavel E. Plyusnin [1], Lyubov G. Bulusheva [1,2] and Alexander V. Okotrub [1,2]**

[1] Nikolaev Institute of Inorganic Chemistry SB RAS, 3 Acad. Lavrentiev Ave., 630090 Novosibirsk, Russia; baskakova@niic.nsc.ru (K.I.B.); artemg@ngs.ru (A.V.G.); plus@niic.nsc.ru (P.E.P.); bul@niic.nsc.ru (L.G.B.); spectrum@niic.nsc.ru (A.V.O.)
[2] Laboratory for Terahertz Research, Tomsk State University, 36 Lenin Ave., 634050 Tomsk, Russia
* Correspondence: o.sedelnikova@gmail.com (O.V.S.)

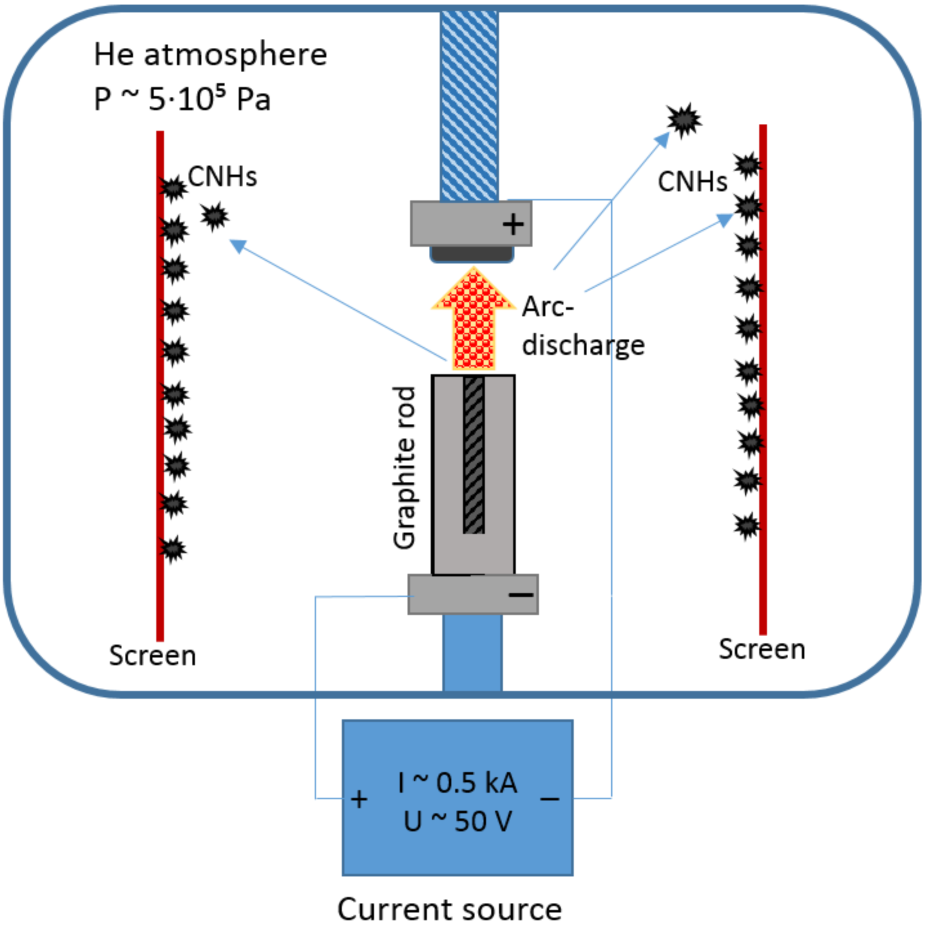


**Figure S1**. Principal scheme of arc-discharge setup. The electrode were placed vertically. An upper movable cathode was made from a water-cooled graphite rod of 60-mm diameter. A graphite anode had a cross-section of 14 × 14 mm and a length of 200 mm. The anode was placed vertically on a water-cooled holder in bottom of the chamber opposite the cathode. Depending on synthesis requirements, the anode was made of solid graphite or graphite rod with cylindrical cavity in central part filled by graphite powder mixed with melamine (2 and 4 wt% of melamine). All syntheses were carried out at arc direct current of 500 A, arc voltage of 50 V and a helium pressure of 5 $10^4$ Pa. Carbon nanohorns were collected from screen placed between electrodes and water-cooled reactor walls made from stainless steel.

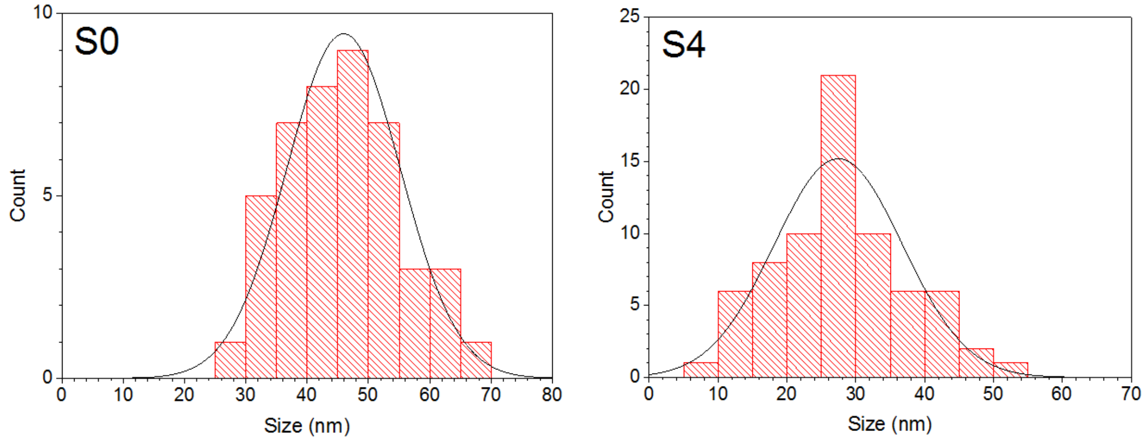


**Figure S2.** Histograms of the size distribution of “bud-like” CNHs in S0 and S4 samples.

Figure S2 show the diameter distributions of bud-like CNHs for S0 and S4 samples. They were obtained from TEM examination of 5 images (100 nm bare) for each structures.

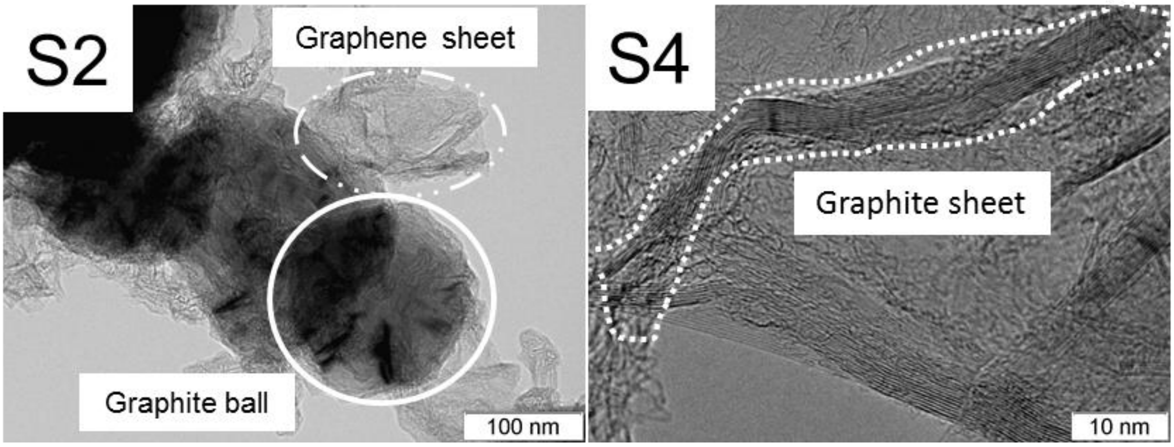


**Figure S3**. TEM images of graphene and graphite sheets found in S2 (**a**) and S4 (**b**) samples.

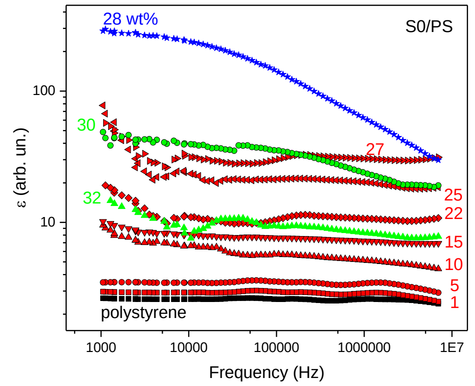


(a)

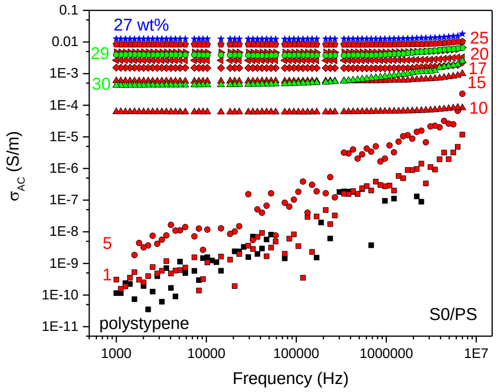


(b)

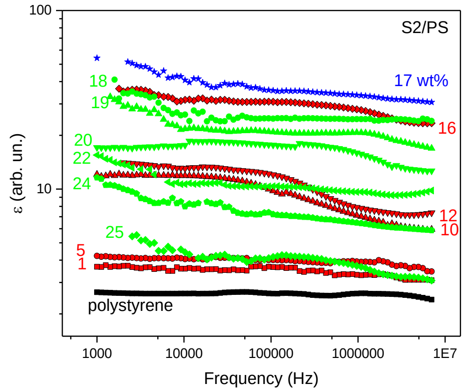


(c)

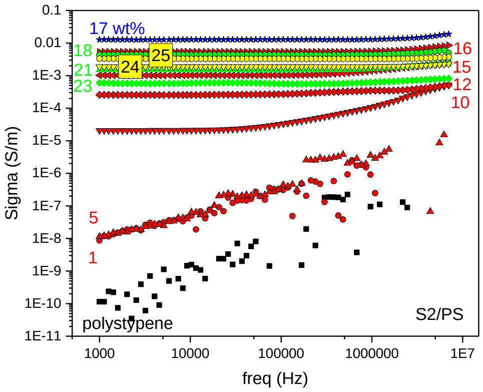


(d)

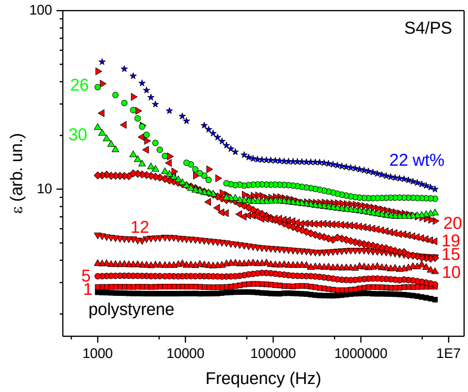


(e)

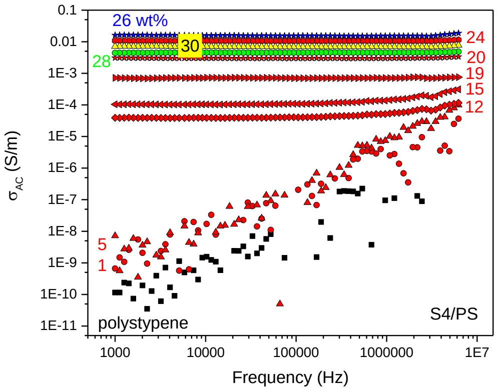


(f)

**Figure S4.** AC permittivity and conductivity for composites containing 1-32 wt% of S0 (**a** and **b**), S2 (**c** and **d**), and S4 (**e** and **f**).

Black symbols correspond to polystyrene (PS) values. The AC permittivity and conductivity increase with loading (red symbols in Figure S4) and rich the maximal value at some critical filler content (blue symbols in Figure S4). Further increase of loading decreases the permittivity and conductivity (green symbols in Figure S4). After the conductivity drop, composites with 24 and 25 wt% of S2 and 30 wt% of S4 demonstrate further conductivity enhancement (yellow symbols in Figure S4).
